# Non‐coding RNAs in cancer immunotherapy: Predictive biomarkers and targets

**DOI:** 10.1002/ctm2.1425

**Published:** 2023-09-21

**Authors:** Murad Alahdal, Eyad Elkord

**Affiliations:** ^1^ Johns Hopkins All Children's Hospital, St Petersburg Florida USA; ^2^ Department of Oncology Sydney Kimmel Cancer Center School of Medicine Johns Hopkins University Baltimore Maryland USA; ^3^ Department of Applied Biology College of Science University of Sharjah University City Sharjah United Arab Emirates; ^4^ Biomedical Research Center School of Science Engineering and Environment University of Salford Manchester UK

**Keywords:** cancer immunotherapy, cancer vaccine, CAR‐T cells, monoclonal antibodies, ncRNA biomarkers, ncRNA targets

## Abstract

**Background:**

To date, standardising clinical predictive biomarkers for assessing the response to immunotherapy remains challenging due to variations in personal genetic signatures, tumour microenvironment complexities and epigenetic onco‐mechanisms.

**Main body:**

Early monitoring of key non‐coding RNA (ncRNA) biomarkers may help in predicting the clinical efficacy of cancer immunotherapy and come up with standard predictive ncRNA biomarkers. For instance, reduced miR‐125b‐5p level in the plasma of non‐small cell lung cancer patients treated with anti‐PD‐1 predicts a positive outcome. The level of miR‐153 in the plasma of colorectal cancer patients treated with chimeric antigen receptor T lymphocyte (CAR‐T) cell therapy may indicate the activation of T‐cell killing activity. miR‐148a‐3p and miR‐375 levels may forecast favourable responses to CAR‐T‐cell therapy in B‐cell acute lymphoblastic leukaemia. In cancer patients treated with the GPC3 peptide vaccine, serum levels of miR‐1228‐5p, miR‐193a‐5p and miR‐375‐3p were reported as predictive biomarkers of good response and improved overall survival. Therefore, there is a critical need for further studies to elaborate on the key ncRNA biomarkers that have the potential to predict early clinical responses to immunotherapy.

**Conclusion:**

This review summarises important predictive ncRNA biomarkers that were reported in cancer patients treated with different immunotherapeutic modalities, including monoclonal antibodies, small molecule inhibitors, cancer vaccines and CAR‐T cells. In addition, a concise discussion on forthcoming perspectives is provided, outlining technical approaches for the optimal utilisation of immunomodulatory ncRNA biomarkers as predictive tools and therapeutic targets.

## Graphical abstract

- ncRNAs in liquid biopsies from cancer patients treated with immunotherapies can be predictive biomarkers.
- ncRNAs function as context‐dependent biomarkers in cancer tissue and immune cells.
- Merging therapeutic ncRNAs with immunotherapy amplifies response and therapeutic impact in cancer patients.

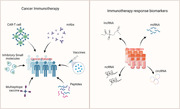

## 1 INTRODUCTION

Cancer immunotherapy works by stimulating the body's natural immune mechanisms to target specific antigens within cancers.^1^ This approach encompasses various techniques, including cell‐based immunotherapy, monoclonal antibodies (mAbs), vaccines, immune checkpoint inhibitors (ICIs) and gene regulatory tools such as coding and non‐coding RNAs (ncRNAs).^2^, ^3^ By fostering immune responses directed at particular targets within cancer tissue, systemic immune reactions can either eliminate or impede the development and functions of malignant cells, leading to enhanced survival rates.^4^, ^5^, ^6^, ^7^ Thus, employing immunotherapy approaches inspired researchers in the clinical treatment of cancer to achieve longer survival and complete recovery. For decades, immunotherapeutic mAbs were used as magic bullets that trigger cytotoxic immune cell responses against tumour cells.^8^, ^9^ Over 100 mAbs were approved by the US Food and Drug Administration against cancer and other immune diseases.^10^, ^11^ Some of the therapeutic mAbs were recruited to stimulate T‐cell activation and some to facilitate antigen presentation to induce adaptive immune responses.^12^, ^13^, ^14^ Despite the promising improvement in the overall survival (OS) of some cancer patients, a significant subset of patients exhibit resistance to mAb therapy due to different reasons, such as inherent factors and the heterogeneous nature of tumours.^15^, ^16^, ^17^

Another promising approach of immunotherapy, the ‘cancer vaccine’, has been explored with the intent of preventing or restraining the growth of diverse cancer types.^18^ Although cancer vaccines have shown limited clinical progress, scientists have recently reported that the trajectory of cancer vaccines expects them to become standard anti‐tumour immunotherapies.^18^, ^19^ This transformation is fueled by the increased identification of tumour‐specific antigens and the promising outcomes of newly developed vaccines in clinical settings.^20^, ^21^ The recently emerged generation of cell‐based immunotherapy has gathered attention, particularly genetically modified immune cells such as chimeric antigen receptor T‐lymphocytes (CAR‐T) cells, which have demonstrated remarkable efficacy in treating B‐cell malignancies.^22^, ^23^ CAR‐T cells are lab‐engineered T cells that recognise and bind to specific surface antigens on the tumour cells and promote specific anti‐tumour immune response.^24^ However, the level of response to CAR‐T‐cell therapy is still uncertain. Despite the significant progress in the development of immunotherapeutic approaches, the establishment of a standard approach to measure and monitor response to immunotherapy remains a critical challenge.^25^ Currently, monitoring changes in differential expression of ncRNAs as sensitive biomarkers can predict response to immunotherapy,^26^ and it is considered an urgent approach to improve clinical outcomes of cancer patients.

ncRNAs are a group of small RNAs that almost do not encode proteins, but they modulate protein production, cellular functions and gene expression.^27^ ncRNAs are currently classified into two groups based on the length of the molecule: long ncRNAs (lncRNAs) consisting of more than 200 nucleotides (nt) in length and small ncRNAs of less than 200 nt, which include microRNAs (miRNAs), small nucleolar RNAs (snoRNAs), ribosomal RNAs (rRNAs), piwiRNAs (piRNAs), transfer RNAs (tRNAs), some circRNAs and others.^28^, ^29^ Overexpression or downregulation of ncRNAs in immune cells was recognized as a specific biomarker of the immune response that can predict cancer progression.^30^ ncRNAs that drive immune regulation and functions are promising biomarkers for measuring the impact of immunotherapy and predicting disease prognosis.^31^ In patients with lung cancer, distinct miRNAs, such as miR‐125b, miR‐21, miR‐99a, miR‐30b, miR‐939, miR‐31, miR‐19b and miR‐15b, were found to exhibit significant dysregulation within T helper 1 cells (Th1) when compared to T cells from healthy donors.^32^ This dysregulation was associated with immune dysfunction and predictive of tumour progression, which can predict tumour progression.^32^ Similarly, in colorectal cancer (CRC), some exosomal miRNAs (Exos‐miRNA) were linked to the downregulation of tumour suppressor genes and tumourigenesis enhancement.^33^ For instance, tumour‐associated macrophage‐derived Exos‐miR‐223 is responsible for suppressing *PTEN* gene expression, which induces drug resistance.^33^ A recent study revealed that plasma miR‐320b, miR‐125b‐5p, miR‐320d and miR‐320c are potential biomarkers predicting the response to anti‐PD‐1 immunotherapy in advanced non‐small cell lung carcinoma (NSCLC).^34^ This study reported that reduced expression of the T‐cell suppressor (hsa‐miR‐125b‐5p) in patients treated with anti‐PD‐1 correlated with a favourable response to immunotherapy due to increased levels of functional T cells.^34^ Therefore, ncRNAs could serve as very sensitive indicators for predicting the response to immunotherapy. In this review, we comprehensively discuss the significance of ncRNAs as biomarkers in cancer patients treated with different immunotherapeutic regimens. Also, the role of these biomarkers in the prediction of positive or negative outcomes of cancer immunotherapy is highlighted.

## 2 PREDICTIVE nRNA BIOMARKERS FOR RESPONSE TO IMMUNOTHERAPY IN CANCER PATIENTS

The significance of ncRNA biomarkers in assessing the performance of immunotherapy has gained substantial importance in recent clinical studies.^35^ The establishment of standardised ncRNA biomarkers for specific subgroups of cancer could provide clinical guidance for immunotherapy utilisation. Within this section, we thoroughly reviewed the reported predictive ncRNA biomarkers for response to immunotherapy in cancer patients.

### 2.1 Predictive ncRNA biomarkers for response to ICIs

Immune checkpoints (ICs) serve as T‐cell immune regulatory markers that contribute to balance T‐cell responses. Through interactions between ICs on T cells and corresponding ligands on cancer cells or antigen‐presenting cells, signals are triggered to restrain T cells from attacking cancerous cells. The advent of ICIs has shown encouraging clinical outcomes, as they facilitate the response to anti‐tumour drugs.^36^ The dramatic development of ICIs offers hope for extended, long‐term survival among patients grappling with metastatic cancers. These innovations serve to augment clinical responses, paving the way for improved outcomes.^37^ Long‐term use of ICIs, however, without early monitoring approaches, may raise the risk of adverse drug reactions such as immune cytotoxicity and organ failure.^37^ Thus, there is an urgent need for validating new molecular biomarkers that monitor the response to ICIs in cancer patients. A recent report highlighted the importance of using ncRNAs as crucial indicators that can predict early responses to ICIs.^38^

A recent study performed to assess lncRNA biomarkers in 1533 NSCLC patients treated with ICIs revealed that lncRNA signature can significantly predict a better response to ICIs, longer OS and an increase in tumour‐infiltrating immune cells.^39^ In this study, the lncRNA signature within the transcriptional profiles of patients afflicted with NSCLC was investigated. These profiles were then compared to the clinical profiles of 187 NSCLC cell lines and 115 immune cell lines. This research revealed significant changes in some ncRNA levels that were linked to high ICs compared to low ICs expression. Deep analyses of these changes determined the correlation between lower IC expression, changes in ncRNA levels and good responses to immunotherapy. In another study, miRNA profiling of NSCLC patients with stage IV disease treated with anti‐PD‐1 revealed that 27 sera miRNAs showed significant changes (22 overexpressed and five downregulated miRNAs). Importantly, the increased levels of miR‐138‐5p, miR‐200, miR‐93, miR‐27a, miR‐34a, miR‐424, miR‐28, miR‐193a‐3p, miR‐106b and miR‐181a in the periphery of patients treated with anti‐PD‐1 were significantly associated with a good response to the treatment.^38^ A clinical investigation examined the RNA‐seq data of tumour samples collected from 348 individuals participating in the IMvigor210 trial who had bladder cancer, as well as from 71 patients with melanoma who were undergoing anti‐PD‐1 therapy. This study revealed that alterations in the expression levels of lncRNAs were associated with an augmentation in cytotoxic T lymphocytes (CTLs) within the tumour tissues.^40^ The changes in lncRNA were linked to significant differences in the rates of OS.^40^ Another study reported lncRNA‐NKILA as a metastasis predictive ncRNA biomarker. A high level of lncRNA‐NKILA was linked to anti‐PD‐1 (pembrolizumab) resistance in patients with triple‐negative breast cancer (TNBC).^41^ The study elaborated that increased lncRNA‐NKILA expression increased K48‐polyubiquitination‐mediated degradation, which mediated degradation of antigen peptide‐loading complex and the intrinsic tumour suppressor genes (Rb and p53), leading to resistance to anti‐PD‐1 drugs and tumour metastasis. Importantly, blockade of lncRNA‐LINK‐A improved CD8^+^ T‐cell infiltration in a mouse model of TNBC, suggesting that lncRNA‐NKILA is a promising predictive biomarker of drug resistance and poor prognosis as well as a potential target for increasing sensitivity to anti‐PD‐1 in patients with TNBC. Another clinical study in melanoma patients with stage IV treated with nivolumab or ipilimumab (anti‐PD‐1) reported the overexpression of different circulating Exos‐miRNAs, such as miR‐155, miR‐146a, miR‐125b, let‐7e, miR‐100, miR‐125a, miR‐99b and miR‐146b that may predict response to immunotherapy.^42^ Increasing levels of these miRNAs were associated with a weak response to anti‐PD‐1 drugs and shorter OS.^42^ Similarly, a study was performed to identify circulating predictive miRNA biomarkers in lung cancer patients for evaluating OS of patients treated with nivolumab and revealed that seven miRNA biomarkers (miR‐411‐3p, miR‐215‐5p, miR‐493‐5p, miR‐495‐3p, miR‐548j‐5p, miR‐93‐3p and miR‐494‐3p) were associated with prolonged OS.^43^ Serum samples obtained from melanoma patients stages I, II and III showed that levels of miR‐150 can predict disease recurrence.^44^ An animal model validation revealed that miR‐150 and miR‐151‐5p decreases in directly was linked to PD‐1^high^ CD4^+^ T cells but blocking PD‐1 can increase the levels of these biomarkers, implying that they could be useful as biomarkers for response to anti‐PD‐1 drugs.^45^, ^46^ A recent study found that miR‐33a overexpression linked to the low levels of PD‐1 and Cytotoxic T‐lymphocyte‐associated antigen 4 (CTLA‐4) in low‐grade and early‐stage lung cancer patients,^47^ suggesting that miR‐33a could serve as a biomarker to predict the effectiveness of anti‐PD‐1 and anti‐CTLA‐4 drugs in lung cancer patients. A recent bioinformatic study analysed clinical data of 865 renal cell carcinoma (RCC) patients and reported that miR‐374c, miR‐6718 and miR‐1269b were upregulated in tumour tissues, which was associated with ICs overexpression, particularly lymphocyte activation gene 3 (LAG‐3).^48^ Increased levels of these miRNAs predict resistance to ICIs in RCC patients and poor prognosis. A preclinical study used glioma‐bearing mice linked the reduced levels of miR‐16‐1 and miR‐15a with an activation of CD8^+^ T cells and downregulation of TIM‐3, LAG‐3 and PD‐1.^49^ In vitro studies showed that blockade of miR‐15a/16‐1 decreased the expression of TIM‐3, LAG‐3 and PD‐1 and enhanced functional CD8^+^ T cells by increasing the expression of mTOR signalling pathway.^49^, ^50^ These findings suggest that miR‐15a/16‐1 as biomarkers of resistance to ICIs in glioma and potential therapeutic targets for improving the sensitivity to ICIs. An interesting study investigated the role of adenosine deaminase acting on RNA‐1 (ADAR1) in melanoma immunomodulation. The study reported decreased levels of ADAR1 in the metastatic transition of melanoma, which enhances biogenesis of miR‐222. This miRNA targets intercellular adhesion molecule 1 and consequently induces melanoma immune resistance to immunotherapy.^51^ High levels of miR‐222 were detected in the melanoma biopsies of ipilimumab (anti‐CTLA‐4) non‐responded patients. The study revealed that miR‐222 is a promising biomarker for assessing the response to anti‐CTLA‐4 drugs in metastatic melanoma.

The link between peripheral circulating exosomal miRNAs and the response to immunotherapy has also been investigated in several studies. Screening of sera obtained from 30 melanoma patients compared to 30 healthy individuals demonstrated significant differential expression of Exos‐miR‐532‐5p and Exos‐miR‐106b. The expression of these miRNAs was higher in melanoma patients than in healthy individuals with 92% sensitivity. The level of expression distinguished between patients with metastasis, stage I–II and those with stage III–IV. Interestingly, levels of exosomal miR‐532‐5p and miR‐106b were significantly declined in patients treated with anti‐PD‐1 (pembrolizumab).^52^, ^53^ The study concluded that exosomal miR‐532‐5p and miR‐106b are promising diagnostic and predictive biomarkers for immunotherapy in clinical settings. Another study reported that overexpression of miR‐1972 and miR‐4502 in the serum of melanoma patients treated with anti‐PD‐1 predicts resistance to anti‐PD‐1 and development of metastatic melanoma.^54^ Altogether, screening of circulating miRNA biomarkers in cancer patients treated with immunotherapy has the potential to identify robust predictive biomarkers that can improve clinical monitoring of responses to ICIs.

**FIGURE 1:**
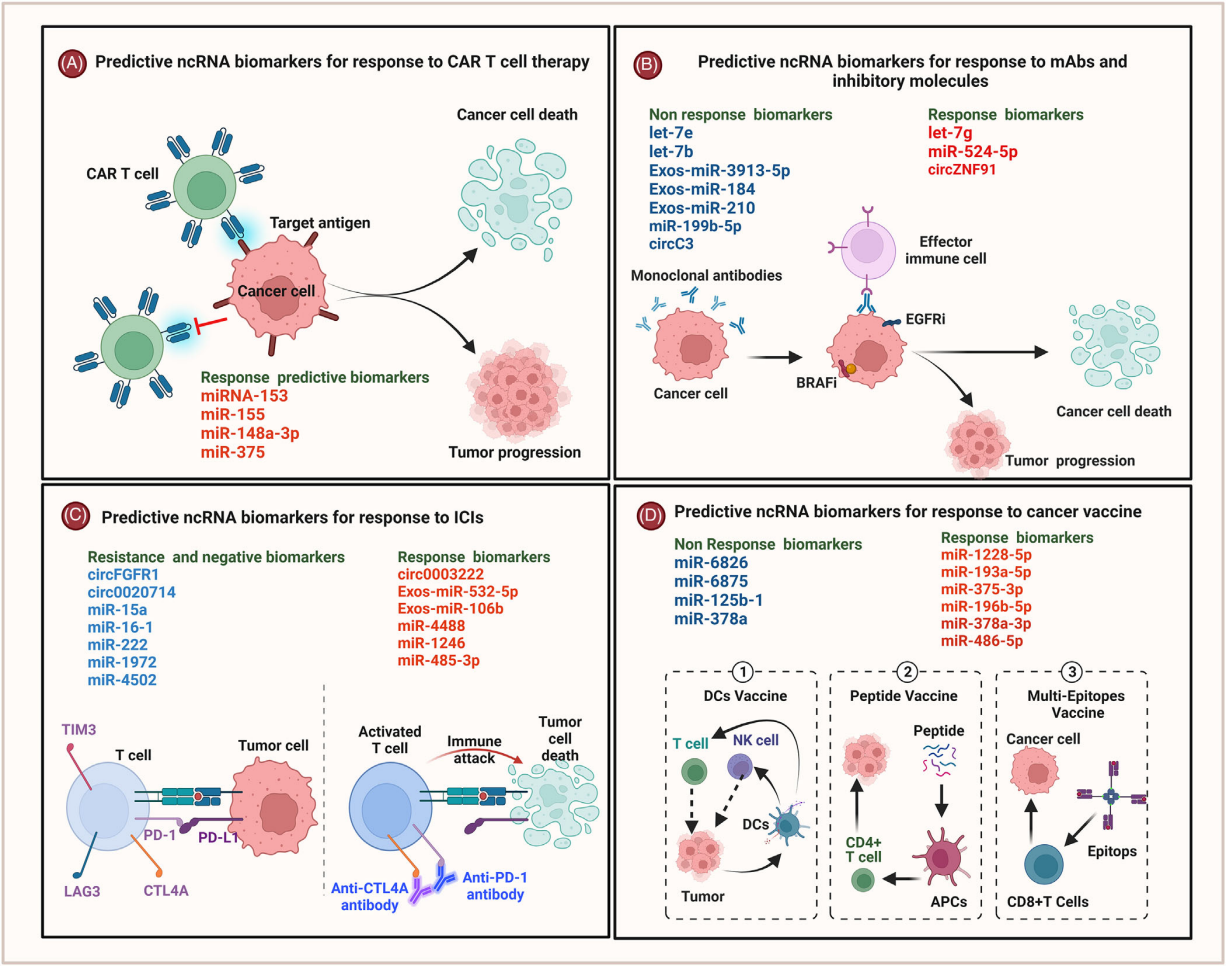
Predictive non‐coding RNA (ncRNA) biomarkers of response to cancer immunotherapy. A schematic diagram shows examples of ncRNAs involved in the response to immunotherapeutic agents. (A) ncRNA biomarkers for predicting response to chimeric antigen receptor T lymphocytes (CAR‐T) cell therapy; (B) ncRNA biomarkers for predicting response to monoclonal antibodies (mAbs) and inhibitory molecules; (C) ncRNA biomarkers for predicting response to immune checkpoint inhibitors (ICIs); and (D) ncRNA biomarkers for predicting response to cancer vaccines. Cancer vaccine types in cancer patients include dendritic cell (DC) vaccine, peptide vaccine and multi‐epitopes vaccine.

Furthermore, circRNAs were also investigated in some clinical studies as biomarkers predicting resistance to ICIs. As presented in Table 1 and Figure 1, elevated circFGFR1 in NSCLC patients is linked to anti‐PD‐1 resistance. In vitro studies elaborated that the anti‐PD‐1 resistance is attributed to circFGFR1's role as a sponge‐like molecule, binding to miRNA‐381‐3p, which increases chemokine receptor 4 (CXCR4) expression and cancer growth.^55^ Another study found a significant link between high levels of hsa‐circ0003222 in tumour tissues and resistance to anti‐PD‐L1 in NSCLC patients.^56^ In vitro experiments determined that targeting hsa‐circ0003222 reduced tumour cell proliferation, migration, invasion and stemness‐like properties via downregulation of PHF21B, which increased tumour suppressor miR‐527 levels. In addition, silencing of hsa_circ_0003222 in the NSCLC mouse model directly contributed to increased sensitivity to anti‐PD‐1 therapy. A recent study investigated the expression levels of circRNAs in NSCLC tumour tissues, compared to adjacent normal tissues and cell lines and found that high levels of hsa‐circ0020714 in NSCLC patients are linked to a poor prognosis and anti‐PD‐1 resistance.^57^ In vitro experiments validated hsa‐circ0020714 as endogenous sponge of miR‐30a‐5p, which induces *SOX4* expression and anti‐PD‐1 resistance. These findings elaborate that hsa‐circ0020714 is a promising biomarker for monitoring the response to anti‐PD‐1 and a potential therapeutic target in NSCLC patients. In conclusion, differential expression of ncRNAs in biological specimens of cancer patients can predict the response to ICIs with high efficiency, which encourages researchers to work on standardising this tool for improving clinical monitoring for timely interventions.

**TABLE 1:** A list of potential non‐coding RNA (ncRNA) biomarkers that predict response to cancer immunotherapy.

Cancer type	ncRNA	Sample type	Expression and predictive function	Ref.
NSCLC	miR‐320b, miR‐125b‐5p, miR‐320d and miR‐320c	Plasma	Downregulation/predicting the effectiveness of anti‐PD‐1 therapy	^34^
	miR‐138‐5p, miR‐200, miR‐93, miR‐27a, miR‐34a, miR‐424, miR‐28, miR‐193a‐3p, miR‐106b and miR‐181a	Blood	Upregulation/biomarkers of good response to anti‐PD‐1 therapy	^38^
	Exos‐miR‐3913‐5p, Exos‐miR‐184 and Exos‐miR‐210	Blood	Upregulation/biomarkers of resistance to Osimertinib	^60^, ^61^
	miR‐125b‐5p	Plasma‐Exos	Downregulation is associated with T‐cell activation and good prognosis	^34^
	circ0003222	Tumour tissue	Overexpression/predicting good response to anti‐PD‐1 therapy	^56^
	circ0020714	Tumour tissue	Overexpression is associated with poor prognosis and resistance to anti‐PD‐1 antibodies by binding to miR‐30a‐5p	^57^
	circZNF91	Plasma	Upregulated in patients treated with EGFR inhibitor (gefitinib) and showed good response	^68^
	circ0002130	Plasma	Upregulated in patients treated with EGFR inhibitor Osimertinib (AZD9291) and showed good response	^69^
Lung cancer	miR‐33a	Serum	Overexpression was associated with low levels of PD‐1 and CTLA‐4 antibodies	^47^
	circFGFR1	Plasma	Binds to miR‐381‐3p leading to resistance to anti‐PD‐1 therapy	^55^
	miR‐411‐3p, miR‐215‐5p, miR‐493‐5p, miR‐495‐3p, miR‐548j‐5p, miR‐93‐3p and miR‐494‐3p	Blood	Overexpression/forecasting a good response to anti‐PD‐1 (nivolumab) therapy and improved OS	^43^
Melanoma	miR‐155, miR‐146a, miR‐125b, let‐7e, miR‐100, miR‐125a, miR‐99b and miR‐146b	Tumour cells Exos	Upregulation/biomarkers of weak responses to nivolumab or ipilimumab	^42^
	miR‐150 and miR‐151‐5p	T cells	Overexpression is associated with efficient blocking of PD‐1	^45^, ^46^
	miR‐222, miR‐1292, miR‐23a‐star and miR‐140‐5p	Tumour tissue	Upregulation is associated with no response to anti‐CTLA‐4 (ipilimumab)	^51^
	Exos‐miR‐532‐5p and Exos‐miR‐106b	Plasma	Downregulation/biomarkers for response to pembrolizumab (anti‐PD‐1)	^130^
	miR‐1972 and miR‐4502	Serum	Overexpression in patients with metastatic melanoma who did not respond to anti‐PD‐1 therapy	^54^
	Exos‐RN7SL1	Tumour cells	Upregulation/enhance CAR‐T‐cell effectiveness	^131^
	miR‐524‐5p and miR‐4488	Tumour cells	Expression is associated with a good response to BRAF and MAPK inhibitors	^62^, ^64^
	miR‐199b‐5p	Tumour biopsies and plasma	Recurrence of melanoma is associated with the expression of this biomarker	^63^
Colorectal cancer	miRNA‐153	Tumour biopsy	Overexpression/ predicting tumour invasiveness but other reports noticed an activation of CD8^+^ T cells when miR‐153 was combined with CAR‐T‐cell therapy	^76^, ^77^
	miR‐6826 and miR‐6875	Plasma	Overexpression predicts low response to cancer vaccines	^87^
	miR‑196b‑5p, miR‑378a‑3p and miR‑486‑5p	Cancer tissue	Predict the efficiency of HLA‑A*2402 peptide cancer vaccine	^88^
	miR‐125b‐1 and miR‐378a	Cancer tissue	Upregulation is associated with low OS in patients treated with peptide vaccines	^89^
Renal cell carcinoma	miR‐374c, miR‐6718 and miR‐1269b	Tumour tissue	Overexpression is associated with increased LAG‐3 and poor prognosis	^48^
Ovarian carcinoma	miR‐1228‐5p, miR‐193a‐5p and miR‐375‐3p	Serum	Overexpression is associated with a good response to the GPC3 peptide vaccine	^85^
Breast cancer	miR‐155	DC	Predictive biomarker for a good response to DCs vaccine in a murine model	^[^ ^86^

Abbreviations: CAR‐T, chimeric antigen receptor T lymphocytes; CTLA‐4, Cytotoxic T‐lymphocyte‐associated antigen 4; DC, dendritic cell; EGFR, epidermal growth factor receptor; NSCLC, non‐small cell lung carcinoma; OS, overall survival.

### 2.2 Predictive ncRNA biomarkers for response to immunomodulatory small molecules

Inhibitory molecules that possess immunomodulatory effects, such as inhibitors targeting the epidermal growth factor receptor (EGFR) and *BRAF*, have demonstrated promising results. These molecules exert their influence by modifying the levels of circulating lymphocytes, cytokines, and even the expression of ICs in cancer patients.^58^, ^59^ Nonetheless, certain cancer patients exhibit limited response to these crucial inhibitory molecules. Early clinical monitoring of response to such inhibitors needs prior studies identifying ncRNA biomarkers that can predict positive responses.

Recent studies investigated peripheral circulating ncRNA biomarkers in NSCLC patients treated with Osimertinib and found that upregulation of Exos‐miR‐3913‐5p, Exos‐miR‐18 and Exos‐miR‐210 is associated with drug resistance and serves as predictive biomarker for metastasis.^60^, ^61^ In the context of melanoma, one report indicated that the expression of a tumour suppressor miR‐524‐5p in patients treated with BRAF inhibitors (BRAFi) serves as a positive prognostic indicator.^62^ Moreover, among melanoma patients treated with both MEK and BRAFi, elevated levels of miR‐199b‐5p in plasma were correlated with recurrence.^63^ This observation suggests that miR‐199b‐5p could potentially serve as a predictive biomarker for response to BRAF and MEK inhibitors. Additionally, the increased levels of miR‐4488 in plasma of melanoma patients post‐MEK inhibitor treatment indicated drug resistance.^64^ In a recent study, the increased levels of miR‐1246 and miR‐485‐3p in plasma were linked to negative response to BRAFi in melanoma patients.^65^ Likewise, miR‐1246^high^/miR‐485‐3p^low^ predicts negative response to BRAFi.^65^ In CRC patients, an early study investigating miRNA signature in tumour tissues of individuals treated with BRAFi revealed that reduced expression of let‐7e and let‐7b could potentially serve as biomarkers for non‐responders to BRAFi therapy.^66^ However, overexpression of let‐7g indicates good prognosis and longer survival.^67^ A recent study analysed the variations in circRNAs among cancer patients who underwent inhibitory molecule treatment and demonstrated intriguing ncRNA biomarkers that offer early predictions of immune responses and prognoses.^68^ The study used RNA microarray on plasma from NSCLC patients treated with an EGFR inhibitor (gefitinib). They observed a significant upregulation of circZNF91 (circ_0109320) in patients with positive treatment responses. Interestingly an in vitro study performed comprehensive circRNA profiling of NSCLC cell lines (H1975/AZDR and HCC827/AZDR) and demonstrated that circC3 (circ_0002130) was significantly upregulated in cells that exhibited resistance to Osimertinib (AZD9291), an EGFR tyrosine kinase inhibitor.^69^ In summary, ncRNA biomarkers from liquid biopsies or tumour tissues hold significant promise in predicting responses to immunomodulatory inhibitory molecules in patients with cancer.

### 2.3 Predictive ncRNA biomarkers for response to CAR‐T‐cell therapy

In recent years, T‐cell‐based immunotherapy exhibited promising progress in treating cancer patients.^70^, ^71^ However, tumour tolerance is challenging because it reduces active immunosurveillance and induces exhaustion of T cells and NK cells. Tumour cells also recruit immunosuppressive cells to support immune escape and immunotherapy failure. A new generation of genetically modified T cells with chimeric antigen receptors (CARs) was created to reprogram and modulate T‐cell functions in the tumour microenvironment.^72^ These are engineered T cells expressing synthetic immunoglobulin/CARs, combined with a tumour‐targeting anti‐scFv binding domain. This fusion is linked to signalling domains such as CD3ζ, enabling self‐directed T‐cell activation.^73^ CAR‐T‐cell therapy improved the clinical efficacy of treating some aggressive leukaemia and lymphoma and has opened avenues for reprogramming tumour resident T cells to become functional.^71^, ^74^ However, tumour heterogeneity, immunosuppressive challenges and other intrinsic mechanisms have increased the resistance to CAR‐T‐cell therapy.^75^ These challenges encouraged scientists to develop multiple signalling generations of CAR‐T cells. However, other side effects hinder the clinical application of CAR‐T‐cell therapy in many clinical trials due to hyperinflammatory adverse effects and immunotoxicity. Standardising clinical monitoring of response to CAR‐T cells using circulating ncRNA biomarkers could predict positive versus negative responses to CAR‐T‐cell therapy. In this context, recent studies have started to report interesting predictive ncRNA biomarkers in cancer patients treated with CAR‐T‐cell therapy. A study conducted in CRC used epidermal growth factor receptor variant 3 mutation (EGFRvIII) as a target of CAR‐T cells and tested the effect with/without miR‐153.^76^ The study revealed that overexpression of miR‐153 significantly enhanced T‐cell killing capability and downregulated the expression of indoleamine 2,3 dioxygenase 1 (IDO1) in tumour cells.^76^ In contrast, a clinical study that followed up CRC patients for 4 years revealed that mRNA profiling showed that miR‐153 overexpression is significantly associated with increased cancer invasiveness (21/30 patients) and platinum‐based chemotherapy resistance.^77^ The study highlighted an upsurge in miR‐153 levels in human primary CRC and advanced stages compared to normal colonic epithelium. Mechanistic investigations unveiled that miR‐153 indirectly promoted invasiveness by stimulating the production of matrix metalloprotease enzyme 9 and directly by inducing drug resistance through inhibiting the forkhead transcription factor forkhead box O3a (FOXO3a).^77^ By checking miRNA databases (Ex:Targtscan7.2), we found that miR‐153‐5p targets IDO1 and other immunoregulatory genes (TargetScanHuman 7.2 predicted targeting of human IDO1). Hence, we propose that miR‐153‐5p could serve as a context‐dependent biomarker suitable for utilisation in monitoring CAR‐T‐cell responses specifically among CRC patients with EGFR mutations. Other studies reported oncogenic function of miR‐155 in breast and lung cancers.^78^, ^79^ However, a new study integrated miR‐155 into the vector of anti‐CD19 CAR‐T cells and reported an increase in the anti‐tumour functions against lymphoma in vivo and in vitro due to induction of interferon‐γ (IFN‐γ) and cytolytic activity of CAR‐T cells.^80^ These results suggest different roles for miR‐155 in immune cells and tumour cells. In other words, the expression of miR‐155 in tumour tissue is a negative biomarker that predicts poor prognosis and drug resistance, but in T cells, it is a positive biomarker that predicts good response to CAR‐T‐cell therapy. This confirms our assumption that miRNA biomarkers in CAR‐T‐cell‐treated patients need to be considered based on the context.

A recent study analysed the transcriptome and regulatory networks of patients with B‐cell acute lymphoblastic leukaemia (B‐ALL) treated with anti‐CD19 CAR‐T therapy and revealed that the increased levels of miR‐148a‐3p and miR‐375 in patients following anti‐CD19 CAR‐T therapy were associated with good responses.^81^ Upregulation of these miRNAs was associated with cancer suppression and activation of anti‐tumour immunity.^81^ This study also elucidated the upregulation of miR‐27a‐3p following CAR‐T‐cell treatment, indicating its role as a tumour suppressor. These findings substantiate the potential candidacy of miR‐148a‐3p, miR‐375 and miR‐27a‐3p as viable biomarkers for B‐ALL patients undergoing anti‐CD19 CAR‐T‐cell therapy.

In total, the studies exploring ncRNA biomarkers within the context of CAR‐T‐cell therapy are currently limited. This scarcity underscores the impetus for researchers to intensify their focus on these biomarkers both pre‐ and post‐treatment. The work in this direction is supposed to discover biomarkers that can exhibit significant alterations in response to CAR‐T‐cell therapy.

### 2.4 Predictive ncRNA biomarkers for cancer vaccines

Therapeutic cancer vaccines (TCVs) are a promising type of immunotherapy inducing specific responses against cancer cells (see Figure 1).^82^, ^83^ TCVs could induce cancer regression, eliminate minimal residual disease, create long‐lasting anti‐cancer memory and hinder adverse consequences.^21^, ^84^ As listed in Table 1, early studies investigated predictive ncRNA biomarkers to improve the efficacy of TCVs and patients’ OS and reported some biomarkers that could predict the response. In ovarian carcinoma patients, researchers conducted an investigation into circulating serum biomarkers to monitor treatment response following administration of the GPC3 peptide vaccine. Utilising miR‐Seq analysis, they scrutinised 84 serum samples sourced from patients participating in a phase II clinical trial of the GPC3 peptide vaccine. Subsequently, miRNA candidates were identified in 14 patients who exhibited favourable responses to the treatment. These findings were validated across a subset of 10 patients who displayed robust responses in comparison to those with lower response rates.^85^ Importantly, the study identified a notable upregulation of serum miR‐1228‐5p, miR‐193a‐5p and miR‐375‐3p, signifying a significant correlation with positive responses to the GPC3 peptide vaccine. Furthermore, these identified miRNAs demonstrated a high predictive value for treatment response in the context of the GPC3 peptide vaccine, enhancing the potential for tailored patient management strategies.^85^ In a preclinical study of breast cancer using a mouse model, overexpression of miR‐155 in murine dendritic cells (DCs) was linked to a significant response to DC cancer vaccines.^86^ Interestingly, a clinical study screened circulating miRNA biomarkers in the plasma of patients with metastatic CRC treated with HLA‐A*2402 peptide vaccine revealed that miR‐6826 was overexpressed in patients with poor prognosis and metastasis.^87^ In addition, increased levels of plasma miR‐6875 in CRC patients treated with this peptide vaccine predicted low efficacy of the vaccine and low survival.^87^ Another study in CRC patients reported that the upregulation of miR‐196b‐5p plus reduced expression of miR‐378a‐3p and miR‐486‐5p, correlated with negative response to the HLA‐A*2402 peptide vaccine.^88^ Furthermore, microarray analysis of tumour tissues obtained from CRC patients treated with a peptide vaccine and chemotherapy presented that both miR‐125b‐1 and miR‐378a were significantly associated with low OS,^89^ highlighting their potentials as predicative biomarkers. A recent study screened 113 plasma samples from CRC patients to demonstrate predictive biomarkers for CRC recurrence using qPCR and revealed that expression of Exos‐miR‐21 significantly predicted CRC recurrence.^90^ Importantly, low expression of Exos‐miR‐21 in DC vaccines against infections such as visceral leishmaniasis predicted the induction of adaptive immune responses by enhancing the release of interleukin‐12 (IL‐12) by APCs.^91^ These findings suggest that Exos‐miR‐21 levels in the plasma of CRC patients treated with DC vaccines can predict response rates and tumour recurrence. Altogether, circulating ncRNA biomarkers in the blood of cancer patients treated with TCVs have the potential to predict clinical responses, OS and prognosis.

## 3 ncRNAs FUNCTION AS IMMUNOMODULATORS IN PATIENTS TREATED WITH IMMUNOTHERAPY

Immunomodulatory therapies can efficiently induce a balanced anti‐tumour immune response, activation of CTLs, and suppression of tumour growth.^92^ However, limitations of these approaches remain because of adverse events and resistance in some cancer types.^93^, ^94^ Currently, the focus on immunoregulatory ncRNAs for provoking and monitoring specific immune responses has increased in cancer immunotherapy^31^, ^95^ (Table 2). In melanoma cells, knocking down miR‐211 restored melanoma sensitivity to BRAFi by disrupting mitochondrial respiration and rendering cells metabolically vulnerable, which increased immunosurveillance and killing of tumour cells.^96^ Furthermore, melanosomal miR‐211 was linked to an increase in MAPK signalling by targeting IGF2R, which supports tumour invasion and enhances drug resistance.^97^ The decrease in MAPK signalling due to knocking down of miR‐211 was associated with the activation of immune killer cells.^98^, ^99^ Importantly, in pancreatic cancer, expression of Exos‐miR‐203 downregulates the expression of tumour necrosis factor (TNF), Toll‐like receptor 4 (TLR4) and IL‐12 in DCs.^100^ This effect can be linked to the low efficiency of DCs cancer vaccines in pancreatic cancer patients.^101^ A recent study demonstrated that overexpression of circMET (circ0082002) in hepatocellular carcinoma (HCC) induced immunosuppression, which was linked to the tumour recurrence and resistance to anti‐PD‐1 therapy by downregulating the expression of miR‐30‐5p.^102^ Inhibition of miR‐30‐5p affects the stability and function of effector T cells,^103^ suggesting that circMET is a potential therapeutic target for promoting anti‐HCC adaptive immune responses. In NSCLC, overexpression of circ0020714 was significantly connected to anti‐PD‐1 resistance through downregulation of miR‐30a‐5p.^57^ Of note, expression of miRNA‐30a‐5p in tumour enhances immune cell infiltration and improves anti‐tumour immune responses.^104^ Another study reported that circ‐CELF1 was elevated in NSCLC.^105^ It binds to miR‐491‐5p and induces resistance to immunotherapy by increasing the expression of *EGFR* gene.^105^ Previous literature reported the significant role of miR‐491 in the regulation of CD8^+^ and CD4^+^ T‐cell proliferation and apoptosis by decreasing the expression of IFN‐γ by targeting cyclin‐dependent kinase 4, the transcription factor T‐cell factor 1 and the anti‐apoptotic protein B‐cell lymphoma 2‐like 1.^106^ Thus, miR‐491 could be a potential immunomodulatory biomarker in cancer immunotherapy. In the same context, circFGFR1 was high in NSCLC, and its high levels downregulated the expression of miR‐381‐3p, and thus induced anti‐tumour immunotherapy resistance by upregulating the expression of CXCR4.^55^, ^107^ Noteworthy, miR‐381‐3p can modulate DC functions by mediating the expression of *CD1c* gene and IL‐10.^108^ In addition, miR‐381‐3p can induce T‐cell differentiation by targeting FOXO1, resulting in the activation of the transcription factors T‐bet and RORγt transcription factors.^109^ Knocking down miR‐381‐3p in vivo showed significant inhibition of inflammatory responses.^110^ By linking these pieces of evidence, miR‐381‐3p could be an important immunomodulatory ncRNA regulating responses to cancer immunotherapy and a predictive biomarker for response in patients treated with cancer immunotherapy.

**TABLE 2:** Immunomodulatory non‐coding RNAs (ncRNAs) in cancer patients treated with immunotherapy.

Cancer type	ncRNA	Immunomodulatory impact in tumour	Ref.
Melanoma	miR‐211	Increases the sensitivity of tumour to the treatment and enhances immune cell response in patients treated with BRAF inhibitors	^96^, ^98^
Pancreatic adenocarcinoma	Exos‐miR‐203	Modulates active functions of DCs by downregulating TNF, TLR4 and IL‐12	^100^
HCC	circMET (hsa_circ_0082002)	Drives immunosuppression by inducing resistance to anti‐PD‐1 antibodies through downregulating the expression of miR‐30‐5p	^102^
NSCLC	circ_CELF1	Induces immunomodulatory and oncogenic effects by inducing the immunosuppression and resistance to anti‐PD‐1 antibodies.	^105^
	circFGFR1	Targets the expression of miR‐381‐3p leading to induce anti‐tumour immunotherapy resistance by upregulating the expression of CXCR4	^55^, ^107^

Abbreviations: CXCR4, chemokine receptor 4; DC, dendritic cell; HCC, hepatocellular carcinoma; IL, interleukin; TLR4, Toll‐like receptor 4; NSCLC, non‐small cell lung carcinoma; TNF, tumour necrosis factor.

## 4 PROMISING USE OF IMMUNOREGULATORY NCRNAS AS TARGETS FOR IMMUNOTHERAPY

**FIGURE 2:**
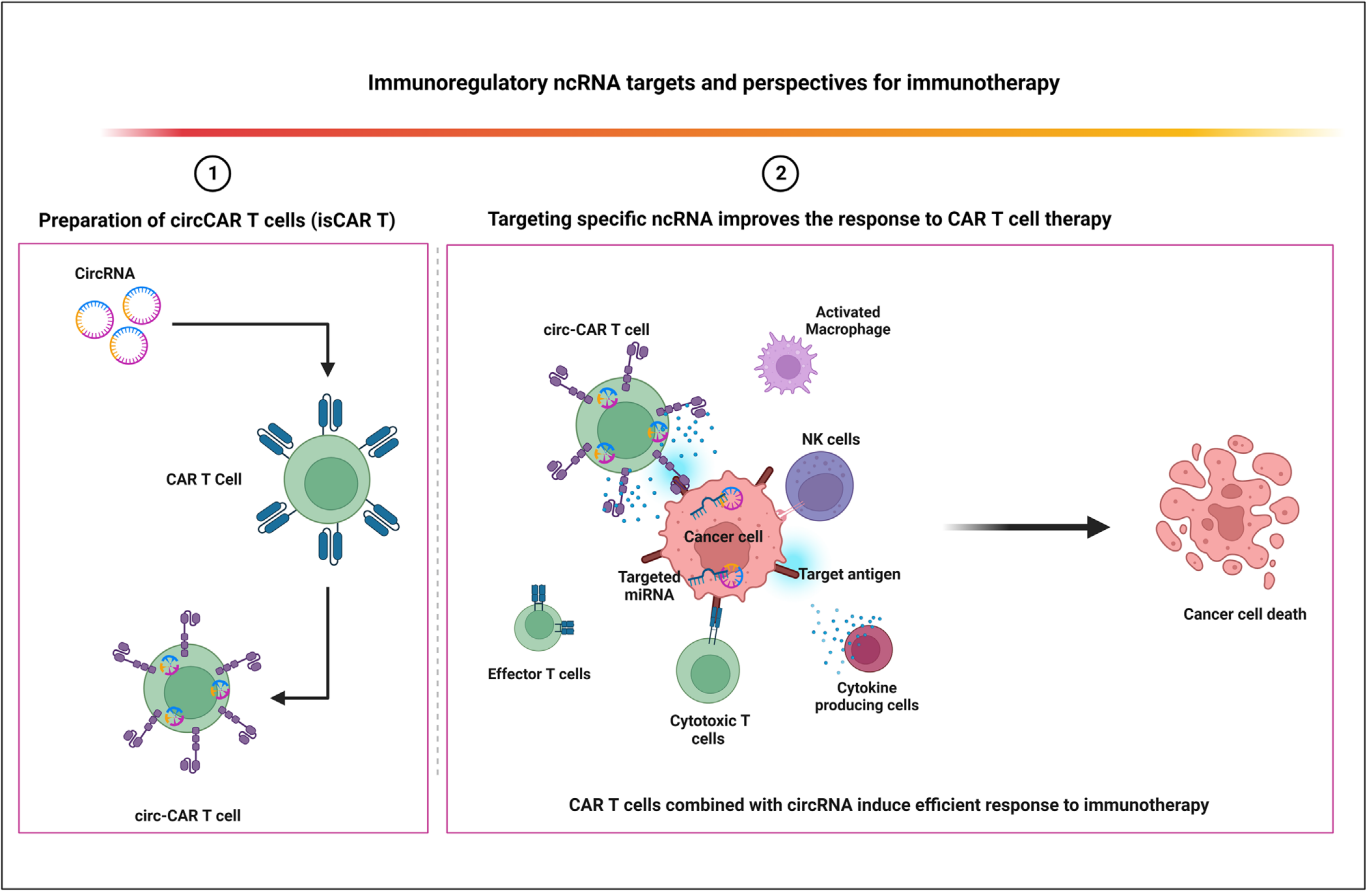
Potential impact of combination between chimeric antigen receptor T lymphocytes (CAR‐T) cell and immunoregulatory non‐coding RNA (ncRNA). A schematic diagram illustrates the potential impact of combining CAR‐T cells and circRNA for improving the response to CAR‐T‐cell therapy. As presented in section (1), circRNAs can combined with CAR‐T cells construction by cloning circRNA in the CAR‐Vector or transfecting CAR‐T cells with the plasmid of circRNA candidate. Section (2): the combination of CAR‐T cell with immunoregulatory ncRNA could improve not only the function of CAR‐T cells but also the response of tumour‐infiltrating immune cells such as effector T cells, cytotoxic T cells, natural killer (NK) cells and activated macrophages besides improving the susceptibility of cancer cells to immunotherapy intervention.

Dysfunctionality of CAR‐T cells has recently been reported in several tumours, which could be attributed to immunosuppressive microenvironment besides other factors.^111^, ^112^ Interestingly, a new study demonstrated that CAR‐T cells in some cancer patients have been forced to express ICs, which induced exhaustion.^113^ A gene‐blocking strategy in CAR‐T cells by ncRNAs was employed to produce modified CD19 CAR‐T cells resistant to exhaustion as reported in modified CAR‐T cells targeting prostate‐specific antigen (PSA).^114^ siRNA‐PD‐1 (siPD‐1) was employed to generate PSA/PD‐1 CAR‐T cells with silenced PD‐1/PD‐L1 pathway compared to CD19/PD‐1 CAR‐T cells. Experimental findings demonstrated that this strategy significantly increased CAR‐T‐cell killing and cytokine release.^114^ This study implies that a new era of immunomodulatory ncRNAs for powering anti‐tumour functions of CAR‐T cells has emerged in the clinical treatment of cancer. As explained in Figure 2, an insertion of circRNA inside CAR‐T cells either by cloning specific circRNA in the contraction of CAR‐Vector or transfecting CAR‐T cells with the circRNA vector for blocking specific suppressor genes in tumour cells or promoting inflammatory responses may improve the response to CAR‐T‐cell therapy, which is suggested to be a promising strategy for solving challenges of exhaustion and low response rates. Recently, it was reported that five doses of Orna's anti‐CD19 isCAR completely eradicated tumours in a xenografted mouse model of acute lymphoblastic leukaemia.^115^ Hence, combination of tumour suppressor ncRNAs and CAR‐T cells would be a novel immunotherapeutic invention in the clinical treatment of cancer, as presented in Figure 2. A recent study reported the link between low expression of circ0064428 in tumour‐infiltrating lymphocytes (TILs) and negative OS in HCC patients.^116^ Overexpression of circ0064428 in TILs can improve immunosurveillance and tumour response to immunotherapy. We prospect a potential efficacy of the combination of circ0064428 with CAR‐T cells for the treatment of HCC patients. Furthermore, transcription of lncRNA‐Malat1 was reported in metastatic NSCLC and other tumours.^117^ Inhibition of Malat1 prevented breast cancer growth and metastasis.^118^ Interestingly, Malat1 was linked to regulatory immune cells infiltrating tumour and to the regulation of cytotoxic T cells, NK cells and macrophages in the TME.^119^, ^120^ Suppression of Malat1 significantly enhanced Th1 and Th2 differentiation and lowered levels of IL‐10 immunosuppressive cytokine.^120^ A study of chromatin‐enriched lncRNAs revealed that Malat1 links with *trans* lncRNAs that boost RNA interactions at gene promoters.^121^ Furthermore, Malat1 promotes terminal effector and terminal effector memory cell differentiation by increasing H3K27me3 deposition at a number of memory cell‐associated genes by direct interaction with Ezh2.^121^ Hence, combination of siMalat1 with CAR‐T cells could be a potential strategy for the treatment of breast cancer.

A recent study reported that lncRNA‐MIR100HG‐derived miR‐100 and miR‐125b induce resistance to EGFRi in CRC patients due to targeting Wnt5/β‐catenin negative regulators.^122^ Blocking lncRNA‐MIR100HG, miR‐100 and miR‐125b in combination with EGFRi could improve response to immunotherapy in CRC patients. In another study, expression of circ0020397 promoted the expression of PD‐L1 and telomerase reverse transcriptase in CRC cells by antagonising miR‐138 suppression of cell growth.^123^ Targeting circ0020397 is supposed to repress the expression of PD‐L1 and thus alleviate the expression of PD‐1 in tumour‐infiltrating T cells, which increases the chance of an effective response to immunotherapy. Furthermore, recent studies reported that expression of circCDR1‐AS, circ‐CPA4 and circ0000284 in several types of cancer, such as CRC, NSCLC, liver and pancreatic cancer, are associated with PD‐L1 expression by targeting CMTM4, let‐7 and miR‐377‐3p.^124^, ^125^, ^126^ Intriguingly, these circRNAs are linked to poor prognosis, tumour progression and deactivation of cytotoxic CD8^+^ T cells. Targeting circCDR1‐AS, circ‐CPA4 and circ0000284 could potentially improve the efficacy of immunotherapy in cancer patients by enhancing CD8^+^ T‐cell responses.

A combination of miRNA with immunotherapy is an attractive strategy, but the complexity of miRNA mechanisms is a serious challenge that needs to be considered carefully in cancer immunotherapy. Because miRNA has context‐dependent functions, it could work as tumour enhancers, but at different background, it could serve as tumour suppressor.^127^ Therefore, the specific mechanism of miRNA in the target cancer should be disclosed before considering this miRNA in immunotherapy. In epithelial ovarian cancer, Exos‐miR‐29a‐3p and Exos‐miR‐21‐5p are released by M2 macrophages and interact with T‐cell subsets, leading to an imbalanced Treg:Th17 ratio and immunosuppression.^128^ Adding siRNA‐miR‐29a‐3p and siRNA‐miR‐21‐5p to immunotherapy regimen could enhance the activation of cytotoxic T cells in ovarian cancer patients and improve the response to anti‐tumour drugs. A similar concept can be used in melanoma, as reported, exosomal miR‐181a/b, miR‐122, miR‐498, miR149 and miR‐3187‐3p are immunomodulators of T‐cell receptor (TCR) signalling that downregulate T‐cell response and induce TNF‐α secretion to suppress CD8 T‐cell activation.^129^ Blocking one of these immunomodulatory miRNAs may significantly enhance activation of cytotoxic CD8^+^ T cells through TCR signalling activation, which is supposed to improve the response to immunotherapy in melanoma patients. Moreover, CAR‐T‐cell therapy can be enhanced by miR‐153 in CRC by inhibiting the expression of IDO1 and increasing the efficacy of T‐cell cytotoxicity.^76^

In summary, exploring ncRNAs to enhance immune responses and optimise immunotherapy holds promise for generating innovative and novel immunotherapeutic approaches in the clinical treatment of cancer. We have highlighted potential ncRNA targets, such as miR‐29a‐3p and miR‐21‐5p in ovarian cancer, circ0020397, miR‐100 and miR‐125b in CRC, and miR‐181a/b, miR‐122 and miR‐498 in melanoma.

## 5 CONCLUSION

Recently, the importance of ncRNAs as predictive biomarkers and therapeutic targets for cancer immunotherapy has gained attention in clinical and preclinical studies. Close monitoring of ncRNA biomarkers provides potential opportunities for early assessment of responses to immunotherapy, patients’ prognoses and cancer relapse. As known, different ncRNAs mediate different pathways in the cancer microenvironment. Monitoring multiple circulating ncRNAs (ncRNome) might thus be a better way to assess immunotherapy response (see graphical Figure). Several clinical studies have reported on predictive ncRNA biomarkers during the course of immunotherapy, such as the upregulation of miR‐138‐5p, miR‐200, miR‐93, miR‐27a, miR‐34a, miR‐424, miR‐28, miR‐193a‐3p, miR‐106b and miR‐181a in NSCLC patients treated with anti‐PD‐1. These biomarkers predicted good prognosis and improved OS. In addition, increased levels of Exos‐miR‐3913‐5p, Exos‐miR‐184 and Exos‐miR‐210 in NSCLC patients treated with Osimertinib predicted drug resistance and metastasis. In patients with B‐ALL treated with anti‐CD19 CAR‐T cells, miR‐148a‐3p and miR‐375 could be potential ncRNA biomarkers for the response to CAR‐T cells. Furthermore, miR‐153 was reported as a potential ncRNA biomarker predicting the response to CAR‐T‐cell therapy in CRC patients. Upregulation of miR‐1228‐5p, miR‐193a‐5p and miR‐375‐3p predicted the response to GPC3 vaccine in patients with ovarian carcinoma. Overexpression of miR‐6826 in the periphery of CRC patients treated with HLA‐A*2402 peptide vaccine predicted poor prognosis and metastasis. Despite the fact that some recent clinical studies have documented various ncRNA biomarkers for evaluating immunotherapy response, the application of molecular biomarker indicators is still a growing field. To our knowledge, no studies have yet explained the technical limits of employing ncRNA biomarkers for monitoring immunotherapy response. Despite the potential shown in various studies with limited case samples, research involving larger case cohorts is required for reliable clinical applications.

The integration of ncRNA biomarker analysis holds promise in uncovering potential immunomodulatory agents and therapeutic targets, enhancing the clinical implementation of cancer immunotherapy. Combining ncRNAs to modulate specific tumour cell signalling pathways alongside immunotherapy interventions has the potential to synergistically amplify treatment responses and ultimately optimise patient prognoses. Moreover, the notable use of miRNA and circRNA biomarkers in clinical studies and limited screening of small and lncRNAs as biomarkers in the circulatory system of cancer patients may be due to the abundant presence of miRNA in liquid biopsies and tumour tissue, facilitated by straightforward screening methods. Importantly, the role of lncRNAs as biomarkers has been explored in many studies, suggesting that lncRNAs can be considered for assessing the response to immunotherapy. We encourage future studies to consider circulating lncRNA biomarkers, which could present a promising sensitivity to immunotherapeutic modalities.

## CONFLICT OF INTEREST STATEMENT

The authors declare they have no conflicts of interest.

## Data Availability

Not applicable.
